# Alpine Cold Vegetation Response to Climate Change in the Western Nyainqentanglha Range in 1972–2009

**DOI:** 10.1155/2014/514736

**Published:** 2014-08-14

**Authors:** Xu Wang, Ziyong Sun, Ai-Guo Zhou

**Affiliations:** ^1^School of Environmental Studies, China University of Geosciences, 388 Lumo Road, Wuhan 430074, China; ^2^Laboratory of Basin Hydrology and Wetland Eco-Restoration, China University of Geosciences, 388 Lumo Road, Wuhan 430074, China; ^3^State Key Laboratory of Biogeology and Environmental Geology, China University of Geosciences, 388 Lumo Road, Wuhan 430074, China

## Abstract

The Tibetan Plateau is regarded as one of the most climatic-sensitive regions all over the world. Long-term remote sensing data enable us to monitor spatial-temporal change in this area. The vegetation changes of the western Nyainqentanglha region were detected by using RS and GIS techniques. And the vegetation coverage was derived by the NDVI-SMA (spectral mixture analysis) methods. An incensement of vegetation was observed in the mountain areas during 1972–2009 with a mean vegetation coverage of 24.87%, 35.89%, and 42.88% in 30/09/1972, 14/09/1991, and 30/08/2009, respectively. The vegetation fraction increased by 18% in the period of 1972–2009. The bin with the elevation between 4400 and 5200 m had the highest vegetation coverage. This may be the result of the mountain effect. Alpine vegetation had a trend to increase and expand to higher altitudes with the climate change in the past 40 years. The variation appears to be associated with an increase in mean temperature of 0.05°C per year and an increase in precipitation of 1.83 mm per year in the growing season of the past four decades. The results provide further evidence of alpine ecosystem change due to climate change in the central Tibetan Plateau.

## 1. Introduction

Due to growth and distribution of plants dependent on the climate conditions and their habitat, vegetation distribution has been shown to be very sensitive to climate and habitat change [1]. Considered as the Third-Pole of the earth, the Tibetan Plateau (TP), with an average elevation of more than 4000 m.a.s.l., is the highest and most extensive highland in the world. Several studies have confirmed an increasing trend of temperature and precipitation on the central TP over the past decades [2–4]. The temperature increase on the TP is greater than the global temperature increase. And the increasing temperature is more pronounced at higher elevations than that at lower elevations [5, 6].

There are large evidences that the cascading effects of rising temperature and loss of ice and snow are giving impacts on water availability (amounts and seasonality), biodiversity (endemic species and predator-prey relations), and livelihoods in Greater Himalayan region during the past decades [7]. More than 80% of glaciers in western China have retreated during past decades [8]. More recent research showed that the alpine grassland had decreased in the context of climatic change in North Tibet [9]. The maximum normalized difference vegetation index (NDVI) correlated significantly with monthly precipitation in the central TP [10]. NDVI had been used a lot in growth status, spatial density distribution, and phenology of vegetation as a good indicator [1]. However, few studies focused on vegetation change to hypsography. In this study, we examined (1) the characteristic of vegetation distribution with regard to elevation and (2) vegetation coverage difference in different elevation zones in the context of climate change in the Western Nyainqentanglha Range (WNR), central Tibetan Plateau.

## 2. Materials and Methods

### 2.1. Study Site

**Figure 1:**
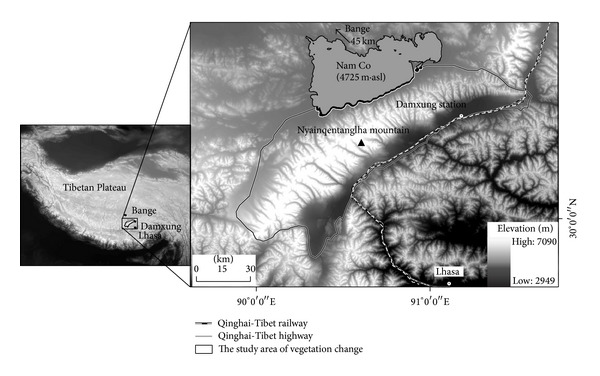
Location of the study area.

The western Nyainqentanglha area is located in the south-eastern centre of the Tibetan Plateau and extends in a NE-SW direction (Figure 1). The average altitude of the WNR is about 5500 m with the highest peak (Mount Nyainqentanglha) reaching the altitude of 7162 m.a.s.l. The study site is bordered by the northern part of the Gangdise Mountains in the SE and by the Nam Co in the NW.

### 2.2. Climate Change

**Figure 2:**
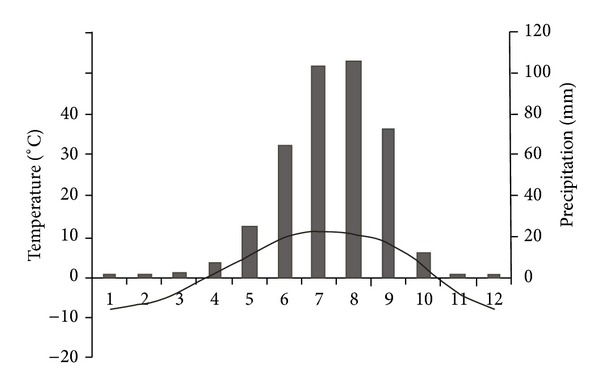
Monthly climatologies of air temperature (1962–2008, curve) and precipitation (1962–2008, columns) for three meteorological stations (Damxung, Bange, and Lhasa). The mean altitude of three meteorological stations is 4500 m.

The mountain ridge is a climate divide. The SE region is located windward to the summer Monsoon. The NW area drains into Nam Co Lake. And the Westerly is prevailing in winter. The mountain climate above 5000 m is little known due to the lack of long-term meteorological records; the climate data can be analyzed from the closest weather stations (e.g., Bange, Damxung, and Lhasa, Figure 1). The area has the maximum monthly mean temperature of 11.5°C in July and minimum monthly mean temperature −7°C in January, and mean annual temperature is 3.3°C. The annual precipitation varies largely between the dry and wet seasons. The annual mean precipitation is 413 mm in recent four decades. Most of the precipitation (80–90%) falls in the warm (rainy) seasons (from May to September) (Figure 2).

**Figure 3:**
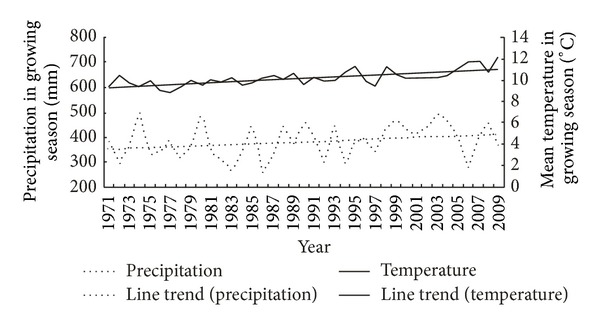
Variability of temperature (1971–2009, solid line) and precipitation (1971–2009, dotted line) in the growing season at three meteorological stations (Damxung, Bange, and Lhasa).

Over the past 40 years, the mean temperature of growing season in the study area showed a significant warming trend and the precipitation showed an increase in fluctuation (Figure 3). The precipitation of the three meteorological stations increased 1.8 mm per year, while the mean temperature of growing season increased 0.05°C per year. And the warming trend in winter is much more obvious than in summer. The mean temperature increased 0.05°C per year from May to September, while it increased 0.059°C per year from December to next February.

Compare to the time period of 1971–1990, there is an acceleration warming in recent twenty years. The linear trend is 0.035°C per year during 1971–1990 and 0.08°C per year from 1990 to 2009, while the linear trend of precipitation is 1.04 mm per year in the period of 1971–1990 and 2.403 mm per year during 1991 and 2009. The climate changes indicate that the air becomes much warmer and moister in this region.

### 2.3. Data and Processing

Landsat MSS/TM and ETM+ scenes were used for the analyses, since the Landsat mission provides long-term remote sensing data back to the middle 1970s. For MSS data, the geometric correction was performed using the autosync model of ERDAS Imagine (Atlanta, GA, USA) with an RMSE of less than 1 pixel. Because there is no suitable calibration factor for the MSS images acquired from different Landsat platforms, it is necessary to intercalibrate the images [11]. The absolute radiometric calibration of MSS products, which puts these images on the same absolute radiometric scale with the TM and ETM+, can be downloaded from the USGS EROS data center. SRTM3 data were downloaded from the CSI-CGIAR data center, version 4, for the characteristic topographic variation of ecoenvironment and then were rescaled to 30 m resolution by polynomial interpolation [12]. The meteorological data was obtained from China Meteorological Data Sharing Service System and China Meteorological Administration.

### 2.4. Methodology

The mountain slopes from bottom to the top of WNR were used as study areas to analyze potential vegetation change. The occurrence of the change in the vertical distribution of the vegetation along the slopes of the mountain range can be attributed to the global warming.

The growing season in the study area is from May to September. Compare to the flat areas, the temperature shows 1-month lag on mountain slopes and the NDVI reaches its maximum 2 months later [13]. Scene selected was based on several criteria: (1) coverage centered on the Nyainqentanglha area, (2) scenes with cloud less than 5%, and (3) approximate coincidence of image acquisition dates. Only three Landsat images satisfied these criteria during the period 1972–2012. The images for fractional green vegetation coverage (FV) were acquired on 30/09/1972, 14/09/1991, and 30/08/2009, respectively.

Vegetation change is a sensitive indicator for climate change [14]. Different methods to derive fractional vegetation were reported including spectral mixture analysis (SMA) [15, 16] and various vegetation indices [17–19]. Taking into account that the main vegetation constituent is alpine grass and that the vegetation change is studied across a large area, the simplified and widely used qualitative NDVI method can be selected [18–20]. Although the NDVI-SMA method overestimated FV in sparsely vegetated areas or barren areas with light soil background [18], it is feasible to compare the relative vegetation change in several different, temporal images.

The following NDVI-SMA model was used to relate FV to NDVI values with a linear mixing model:

(1) $$\begin{array}{c} \text{FV}=\frac{\left(\text{NDVI}-{\text{NDVI}}_{\text{soil}}\right)}{\left({\text{NDVI}}_{\text{gv}}-{\text{NDVI}}_{\text{soil}}\right)},\\ \text{NDVI}=\frac{\text{NIR}-\text{RED}}{\text{NIR}+\text{RED}}, \end{array}$$

where NDVI is the pixel NDVI value, NDVI_soil_ is the bare soil NDVI, and NDVI_gv_ is the dense green vegetation NDVI.

In the WNR, there were few cloud-free Landsat images available for the growing season of the vegetation. As a first step, remaining clouds were masked to assess the mappable area. Other masks included cloud shadow, water bodies, and glaciers. We combined the three masks into a total mask and then applied the mask to each Landsat image before calculating the NDVI values. The pixels of bare soil and dense green vegetation were derived from 2-dimension spectral scatter plots in ENVI 4.8. Finally, the FV values were computed.

A GeoEye-1 high-resolution image acquired on August 30, 2009, the same day as the TM scene (30/08/2009), was used to verify the accuracy of vegetation coverage.

**Figure 4:**
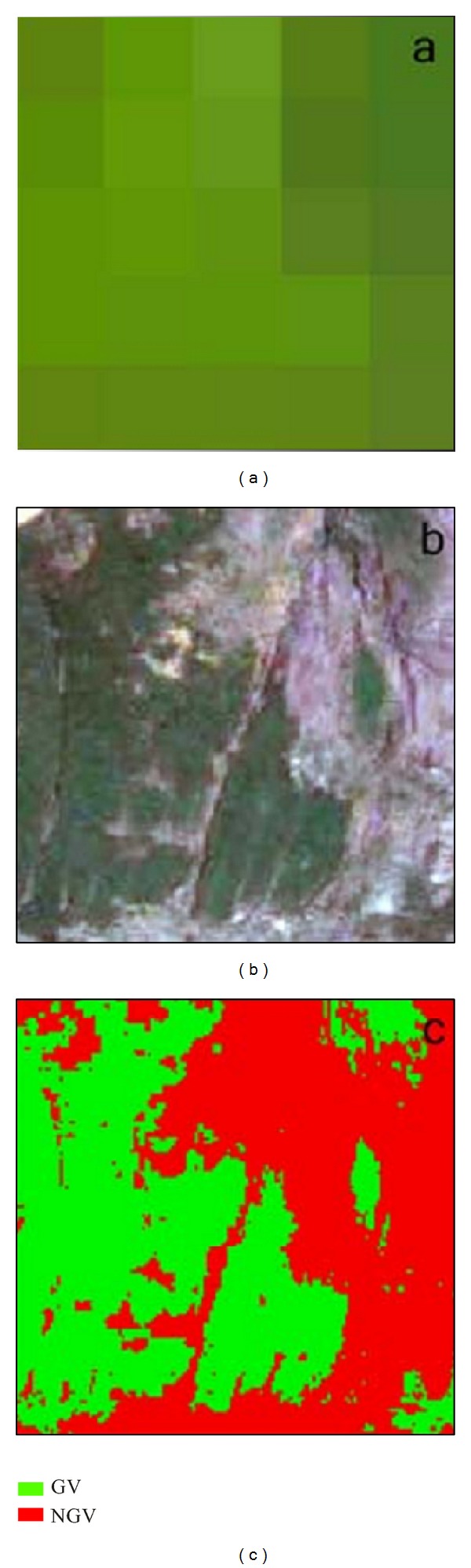
The estimation of actual fractional green vegetation cover (FV) with the 5 × 5 window surrounding each sample TM pixel from the high resolution image. (a) is the 5 × 5 pixel of TM subset with 5-4-1 band combination; (b) is the high resolution subset corresponding to the 5 × 5 pixel; (c) is the classified results of the high resolution subset. GV is the abbreviation for green vegetation, and NGV represents no green vegetation.

We randomly selected 60 plots (5 × 5 pixels) from the TM images and calculated the average FV of each plot (Figure 4). The high resolution GeoEye-1 was used to estimate the actual FV of these sample TM plots and then compared with their predicted vegetation fractions for evaluation of FV calculated by NDVI-SMA. Each high resolution plot was classified into green vegetation (GV) and no green vegetation (NGV) using maximum likelihood classification. Correlation coefficients *R* ^2^ and overall RMSE were 0.89 and 0.054, respectively. This approach was also used to estimate the FV in 1991 and 1972.

## 3. Results

### 3.1. The Fraction of Vegetation Coverage Change

**Figure 5:**
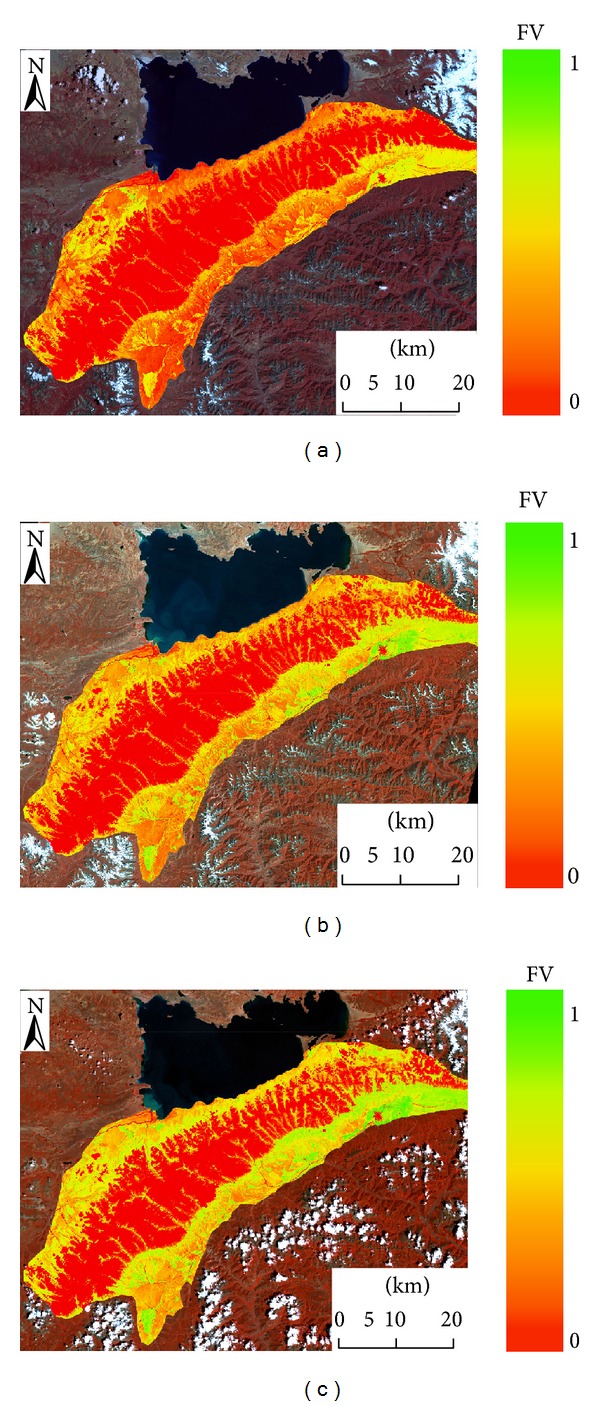
The vegetation fraction maps of the WNR in (a) 1972, (b) 1991, and (c) 2009. The FV values show the percentage of vegetation cover in each pixel (image band composition RGB: 421).

Based on the limited data, the aim of this study was to investigate the characteristic of vegetation distribution with regard to elevation and response of long-term vegetation coverage to climate change in the Western Nyainqentanglha Range (WNR), central Tibetan Plateau. Mountain vegetation is particularly sensitive to climate change, especially at higher altitudes. As one of the most significant indicators of vegetation condition, the fraction of vegetation coverage was obtained from Landsat images as described in the Materials and Methods (Figure 5). A significant vegetation increase can be observed in this area during 1972–2009. The mean fraction of vegetation cover in 30/09/1972, 14/09/1991, and 30/08/2009 was 24.87%, 35.89%, and 42.88%, respectively, and increased by 18.01% in the period 1972–2009. The trend is in line with previous research results in Alps mountains [21].

### 3.2. Characteristic of Vegetation Distribution

**Figure 6:**
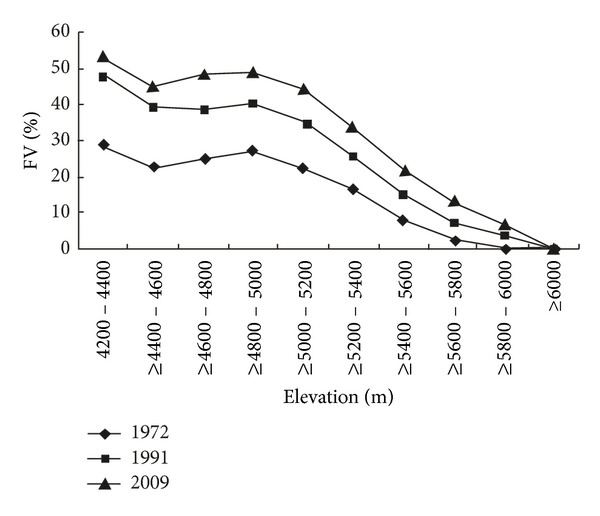
The mean vegetation cover fraction along the altitude gradient in 1972, 1991, and 2009.

The study area was divided into ten bins (each 100 m elevation) from elevation 4200 to 6000 m by using the digital elevation model (DEM). The average vegetation coverage fraction of each bin was obtained in different periods from 1972 to 2009 (Table 1). It is apparent that the vegetation cover decreased gradually with the elevation rise in the areas from 5200 to 6000 m. However, the vegetation coverage is almost stable in 1972, 1991, and 2009 with the elevation from 4400 to 5200 m (Figure 6), suggesting that the precipitation may stabilize at these bins. These indicated that the vegetation distribution was affected by the mountain effect. The highest vegetation coverage zone is the elevation of 4200–4400 m, where it is at the foot of the mountain with gentle slope. There was no vegetation found in the areas with the elevation above 6000 m, suggesting that the elevation from 5800 to 6000 m may be the limit height of vegetation growth in WNR.

### 3.3. The Vegetation Coverage Change Difference in Different Elevation Zones

As can be seen in Table 1, there is an expansion trend of alpine vegetation to higher altitudes in the study area from 1972 to 2009. It is apparent and was noted by Pauli et al. [22] that climate warming is expected to shift species ranges to higher altitudes in mountainous regions. This can be demonstrated in Figure 6, where the friction of vegetation coverage in high bin in 1991 is very close to the lower bin in 1972 at the elevation of 5000 m to 6000 m. The same phenomena can be observed in each zone above 5200 m from 1991 to 2009. It implied that vegetation has an upslope to higher altitudes in higher altitude area. Kelly and Goulden [23] found that the rising of plant species to higher altitudes with 65 m appears to be a consequence of changes in regional climate in Southern California's Santa Rosa Mountains, which is with lower altitude.

**Figure 7:**
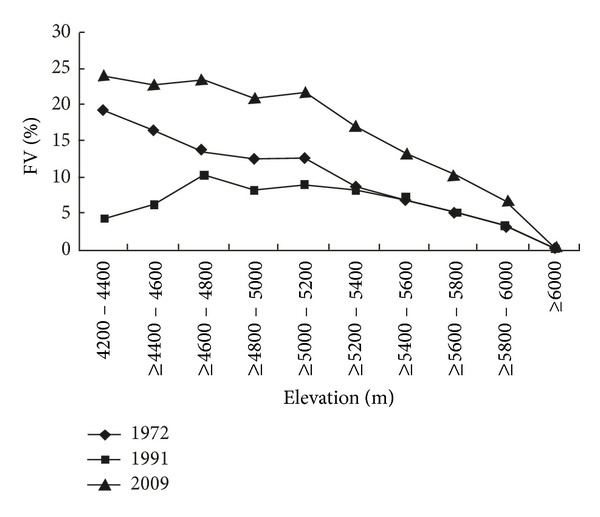
The increased fractional vegetation cover along the ascending elevation during the three periods of 1972–1991, 1991–2009, and 1972–2009.

Figure 7 shows that it is obvious that the increased fractional vegetation cover is relatively lower but nearly the same during 1972–1991 and 1991–2009 with the elevation of 5400 m to 6000 m. It may reflect that the vegetation is sensitive to climate change at higher altitude. Although the rate of warming is speeding up compared to the past decades, the vegetation cover showed lower increase rate during 1991–2009 than that in the period of 1972–1991 (Figure 7). It implied that the increase in vegetation cover is not linearly related to temperature rising.

## 4. Discussion

In mountainous ranges, plant species is sensitive to climate change. Effects of climate change on vegetation have already been detected across Himalayas [7] and Alps [24]. Landsat imagery is an important tool to document historic vegetation coverage and to investigate and analyze its change over large areas. We found a significant vegetation increase of 18.01% during the period 1972–2009 in the remote area of WNR, central Tibetan Plateau. Analyzing the vegetation distribution in elevation zones indicated an expansion of vegetation trend to higher altitude. Our results are in accordance with previous studies. The alpine grassland increased significantly in recent years on the Tibetan Plateau and it corresponded to the increased temperature and precipitation, especially in the central part of the Tibetan [10, 13, 25]. The increasing trend of vegetation was also observed in the core area and southern slope of Mt. Qomolangma Nature Reserve [26].

In fact, the mechanisms of the vegetation change are complex; it is difficult to explain the relationship by using the combination of several ecological factors. Climate change, elevation gradient, soil characteristics, and habitat conditions are the main drivers for vegetation change [27].

### 4.1. Climatic Change

Climate is one of the important factors of vegetation distribution in large scale. The high elevation vegetation is vulnerable to the effects of a warming climate [28].

And the vegetation distribution and variation can reflect the differentiation and climate change in some extent. Generally speaking, the activity of internal metabolism of plant will speed up with the increased temperature in alpine area. Monthly average temperature in this area indicated that temperature reached the peak in the growing season and plants began to grow in May as temperatures rise. The warming trend in Nyainqentanglha is consistent with the trend of global warming.

The study site is located in alpine cold zone and therefore low temperature is the main factor limiting vegetation distribution in maximum vertical altitude. Increased temperature and nutrient decomposition could promote vegetation growth at an alpine zone [7]. And the rising temperature is beneficial to the move up of maximum vertical habitat that suitable for plant growth. The rapid warming trend has a promoting effect on the increase in vegetation coverage on the plateau. Our data provide evidence for the vegetation trend to higher elevations. The trend of increased vegetation coverage is consistent with warming trend in the other area. Increasing temperature and decreasing snowpack result in increased shrub growth and range expansion at rock glacier site in the eastern Sierra Nevada of California [29]. Lenoir et al. [24] showed that climate warming has resulted in a significant upward shift trend in species optimum elevation averaging 29 m per decade from 1905 to 2005 in west Europe. By analyzing the phenological observation data in 2007 and 2008 in this area, the plants flower as early as mid-May till late September. Compared to 2007, the temperature is lower that most flowering and fruiting of plants generally get shorten about 5 d in 2008 [30].

Not only warming but also the change in precipitation plays an important role in determining the impacts of climate change on vegetation [31]. The study site is located in semiarid climate area. The annual rainfall is 409 mm and the precipitation mainly concentrated in the period from May to September, accounting for more than 90% of the total annual precipitation. The wetland vegetation in the northern slope of Mt. Qomolangma Nature Reserve degraded due to the reduced precipitation [26]. The precipitation in the growing season was significantly increased in the western Nyainqentanglha area in the last 2 decades. Interannual variability of plant phenology is more sensitive to precipitation. The earlier rainy season makes phenophase ahead of about 20 d in advance [30]. The increased vegetation cover may partly be attributed to the increasing precipitation in the growing season.

### 4.2. The Mountain Effect on the Vegetation Coverage

Generally, the growth of vegetation was mainly influenced by temperature, precipitation, and light. The temperature decreases with altitude increasing. However, in mountain area, the slope area absorbs more solar radiation and the warming rate is much more significant than lowlands. Also, the current ascends with underlying surface uplift and condenses to precipitation. This causes the precipitation and precipitation intensity to be also subsequently rising with the increase in altitude in the mountainous area and reaches a maximum value at a particular height, which is called a maximum precipitation height. The vegetation coverage fraction is high and stable at an altitude of 4400–5200 m, which may be influenced by the mountain effect.

The spreading of the alpine vegetation and its upslope shift noted that the climate change has significant impacts on the alpine vegetation. Similar phenomena have been widely demonstrated. Lenoir et al. [24] suggested a larger upslope migration trend for species restricted to mountain habitats and for grassy species in West Europe in 1986–2005, comparing to 1905–1985. Some studies also proved that the habitats of shrub and grass tend towards higher elevations with the increase in temperature [32]. Due to the topographic variability, alpine landscapes are likely to be safer places for most species than lowland terrain with climate warming [33]. Yang et al. [14] confirmed that cushion plants increased with elevation in the Sino-Himalayan region by field investigation, and interactions were predominately at high elevation habitats.

### 4.3. Other Factors

Changes in permafrost can significantly affect the alpine vegetation [34]. Although the permafrost change is difficult to monitor by remote sensing data, the vegetation change can be used as an indirect indicator of permafrost change. Many studies have reported that the permafrost is degrading and its limits move uphill due to temperature increase [35, 36]. In general, the greater the vegetation coverage, the lower the heat flux entering the subsurface soil and the lower the thawing rate [34]. Although in the near future the vegetation will increase with the suitable temperature and moist soil on western Nyainqentanglha area, the montane cold vegetation will lose its their suitable habitat with the climate warming in the long run. Gottfried et al. [37] also provide evidence that more warm-adapted species increase and more cold-adapted species decline above the treeline in all major European mountain systems. The glacier retreat and increased number of glacier lakes shifted to higher altitudes [38] indicated the potential habitat for vegetation expansion to high elevations in the area in the future if climate warming continues.

There are still other ecoclimatological factors, such as solar radiation, soil moisture, and snow melt, which have not been explored in this study, but may also account for variability in vegetation growth on the Tibetan Plateau. Thereby, longer-term and denser time series satellite data are needed to investigate interactions at different spatial and temporal scales and to predict the response of land cover to climatic impacts. And detailed explanation has yet to set up automatic meteorological station in a high altitude area for further research and analysis. Less attention was paid to the vegetation composition and vegetation type change in this study. Further studies are required to know more about the biodiversity change in the alpine region under the influence of climate change.

## 5. Conclusions

The central Tibetan Plateau is considered one of the most sensitive regions under the climate change in the world. This study investigated the alpine vegetation coverage change by using Landsat imagery between 1972 and 2009. There was an obvious vegetation increase in the mountain area during 1972–2009. The increasing trend of fraction of vegetation cover agreed with the increase in annual temperature and annual precipitation in growing season. The improvement of temperature and precipitation is the primary factor for the increasing vegetation cover. The vegetation distribution was also affected by the mountain effect.

The research only involves the vegetation coverage distribution and vertical elevation changes. Although in the near future the vegetation will increase with the suitable temperature and moist soil on western Nyainqentanglha area, the montane vegetation will lose its suitable habitat with the climate warming in the long run. Thereby, persistent attention should be paid to the influences of climatic change on this region.

## Figures and Tables

**Table 1:** Distribution and change of the fraction of vegetation cover in each elevation zone of the study area from 1977 to 2010.

Elevation (m)	30/09/1972	14/09/1991	30/08/2009	1972–1991	1991–2009	1972–2009
	FV (%)	FV (%)	FV (%)	ΔFV (%)	ΔFV (%)	ΔFV (%)
>6000	0	0	0	0	0	0
5800–6000	0.17	**3.72**	**7.04**	3.55	3.32	6.87
5600–5800	**2.6**	**7.82**	**13.04**	5.22	5.22	10.44
5400–5600	**8.4**	**15.01**	**21.91**	6.61	6.9	13.51
5200–5400	**16.59**	**25.8**	33.92	9.21	8.12	17.33
5000–5200	**22.34**	35.1	44.29	12.76	9.19	21.95
4800–5000	*27.45 *	*40.20 *	*48.59 *	12.75	8.39	21.14
4600–4800	*25.27 *	*38.59 *	*48.88 *	13.32	10.29	23.61
4400–4600	*22.84 *	*39.23 *	*45.5 *	16.39	6.27	22.66
4200–4400	29.07	48.5	53.04	19.43	4.54	23.97
